# Strain‐Induced Redistribution of Point Defects in ZnO Nanoparticles

**DOI:** 10.1002/smsc.202500642

**Published:** 2026-03-23

**Authors:** Korbinian Aicher, Thomas Berger, Antonios Litovoilis, Ulrich Aschauer, Oliver Diwald

**Affiliations:** ^1^ Department of Chemistry and Physics of Materials Paris‐Lodron University Salzburg Salzburg Austria; ^2^ AMOLF Amsterdam Netherlands

**Keywords:** coke, flexoelectronics, intralattice hydrogen, particles in contact

## Abstract

Powder compaction results in local strains in ZnO nanoparticle ensembles, which alter the abundance of intrinsic defects that determine the electronic structure of the semiconductor. Defect formation energies obtained by density functional theory (DFT) calculations predict that oxygen vacancies (V_O_) and hydroxyl groups substituting oxygen lattice sites (OH_O_) represent the most abundant point defects in ZnO nanoparticles grown by gas‐phase synthesis, where particle nucleation and growth occurs at 1000 K, in oxygen‐poor and—due to hydrocarbons in the precursor solution—hydrogen‐containing atmosphere. Experimentally, we show with vis–near‐infrared diffuse reflectance and electron paramagnetic resonance spectroscopy that uniaxial ZnO nanoparticle powder compaction produces grain‐size dependent and strain‐induced depletion of lattice hydrogen (OH_O_). Related trends can be consistently explained by DFT via stress‐induced destabilization of protonated oxygen, leading to hydrogen release. Moreover, we found that polyaromatic surface carbon covering ZnO grains as extrinsic defects serves as a strain absorber and compensates for the compaction‐induced changes in the concentration of point defects. These findings provide key insights for the defect engineering of particulate ZnO nanostructures in physical contact and are of high relevance for the exploitation of their flexo‐ and piezoelectric activity.

## Graphical abstract

Powder compaction generates local strain effects in ZnO nanoparticles. This alters the abundance of intrinsic point defects and impacts the semiconductor's electronic structure. Such changes were analyzed by a combination of spectroscopy and density functional theory calculations and emphasize the importance of strain effects in the design and processing of particle‐derived functional materials for sensing, piezoelectrics, and photocatalysis.

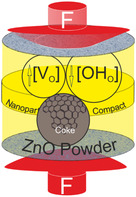

## 1 Introduction

Local forces between contact points and asperities of adjacent ZnO nanostructures can significantly impact their electronic structure and chemical reactivity. Due to its intrinsic piezoelectric and photoelectric properties, ZnO has attracted considerable attention as a flexoelectric material. These interests relate to strain‐induced effects in piezoelectric and photoelectric catalysis [1, 2, 3], or to the performance of ZnO in photodetection devices [4] and sensors [5, 6, 7].

Powders of ZnO particles in different size regimes are a common source for device components in varistors [8], sensors [5, 6, 7], (photo)catalysts [9, 10, 11, 12], and (photo)electrodes [9, 13, 14]. For the fabrication of these devices, powder compaction represents a key manufacturing step. The associated emergence of strain‐induced changes in electronic structure prompts the question of how defects of various kinds do arise or disappear in the course of processing‐related contact phenomena. Since many of the above mentioned applications of ZnO rely on the physics and chemistry of point defects [15, 16] like oxygen vacancies (V_O_), zinc interstitials (Zn_i_), and hydrogen‐related species such as hydroxyls that substitute O^2−^ ions in the ZnO lattice (OH_O_) [17], related knowledge is a prerequisite for an effective defect engineering.

An additional motivation to investigate and rationalize the influence of point defects on reactivity and structure formation in nanocrystalline powders under compaction arises from the field of cold sintering. Cold sintering is a promising energy‐saving alternative to conventional sintering processes, as temperatures not higher than 300°C are required for materials densification. Only recently it has been observed that cold sintering‐induced protonation of the metal oxide lattice promotes the formation of defects and electronic donor states [18].

Spectroscopic analysis—in particular the combined use of (UV–vis–NIR, PL emission) and electron paramagnetic resonance (EPR) spectroscopy [19, 20, 21]—of cold sintered ZnO samples has proven as extremely insightful for the analysis of point defects and chemical species that contribute to the reactivity of the transient phase and determine the densification behavior of the samples [18]. A commonly reported spectroscopic and defect‐related fingerprint in ZnO synthesized via various methods is the electron paramagnetic resonance (EPR) signal at *g* = 1.96. The microscopic origin of this signal has remained a topic of ongoing debate, with both intrinsic and extrinsic defect models proposed [22, 23, 24, 25, 26]. In recent work [17], we have come to the conclusion that this paramagnetic resonance signal corresponds to OH on lattice oxygen sites (OH_O_^0^) and addressed the overlooked role of hydrogen incorporation into ZnO during synthesis and materials processing. A consistent explanation has been put forward to rationalize the emergence of such defects during sintering of commercial ZnO powders [18].

While cold sintering involves both chemical additives and thermal activation, the present study explores whether comparable defect responses can be pinned to mechanical compaction alone, i.e. in the absence of elevated temperatures and a reactive chemical environment. Mechanical compaction introduces local strain and enhances interparticle contact, both of which can influence charge distribution and defect stability. Prior studies on high surface area metal oxides—such as MgO [27], TiO_2_ and BaTiO_3_ [28]—have demonstrated that uniaxial pressing can modify the electronic configuration of anhydrous nanoparticulate systems by promoting charge transfer and/or defect formation. Building on these findings, we hypothesize that semiconducting ZnO nanoparticles undergo substantial electronic changes upon compaction at room temperature, including the generation, annihilation and redistribution of point defects. This work, therefore, focusses on the potential strain‐induced generation, annihilation and redistribution of point defects and aims at decoupling the contributions of strain, particle contact, temperature, and chemical reactivity for knowledge‐based defect engineering.

The effects of mechanical compaction on the electronic and optical properties of ZnO nanoparticle powders are addressed for the first time by using a combined experimental and theoretical approach. EPR and vis–NIR spectroscopy are employed to monitor changes in defectrelated signals and optical absorption, while density functional theory (DFT) calculations provide complementary insights into compaction‐ and strain‐induced modifications. Related insights significantly advance our capabilities in defect engineering of ZnO‐based nanomaterials and related semiconducting materials.

## 2 Methods

### 2.1 Nanoparticle Synthesis and Thermal Annealing

ZnO nanoparticles were produced by chemical vapor synthesis (CVS) and flame spray pyrolysis (FSP).

#### 2.1.1 CVS

For the synthesis of ZnO nanoparticles via metal‐organic chemical vapor synthesis (MO‐CVS), zinc acetate dihydrate (Sigma–Aldrich, ≥99.0%) was subjected to combustion in a flow reactor system in O_2_ atmosphere (p(O_2_) = 15 mbar, flowrate: 650 sccm). Further details about the synthesis conditions can be found in refs. [19, 20, 21, 29].

#### 2.1.2 Flame Spray Pyrolysis (FSP)

Details about the self‐constructed flame spray apparatus and the synthesis conditions can be found in refs. [30, 31].

To prepare the precursor solution, 16.46 g zinc acetate dihydrate (Sigma–Aldrich, ≥99.0%) were dissolved in a solvent mixture comprised of 20 ml methanol (≥99.8%, Sigma Aldrich), 33.4 ml 2‐ethylhexanoic acid (≥99%, Sigma Aldrich) and 39 ml xylene (≥98.5%, VWR Chemicals), yielding a zinc concentration of *c*_Zn_ = 0.75 mol·l^−1^. The mixture was refluxed for 3 h at 323 K using a reflux condenser.

#### 2.1.3 Thermal Annealing

The thermal annealing protocol consists of three sequential steps, as schematically illustrated in the Supporting Information (Figure S1).

In the first step, the ZnO nanoparticle powder is heated up to 473 K at a rate of 2.5 K·min^−1^ under continuous pumping at dynamic high vacuum of *p* < 10^−5^ mbar, followed by a 30‐minute dwell at this temperature. The sample is then cooled to room temperature. In the second step, 650 mbar of molecular oxygen (O_2_ 5.0) are added, and the sample is heated to either 573 or 673 K (depending on the annealing program) using a rate of 5 K·min^−1^. After a dwell time of 30 min, the sample is cooled to room temperature and subsequently re‐evacuated to high vacuum (*p* < 5·10^−5^ mbar). In the third and final step, 650 mbar of molecular oxygen (O_2_ 5.0) are added, and the sample is heated to either 673 or 873 K (depending on the annealing program) using a rate of 5 K·min^−1^ and a dwell time of 60 min. The sample is then cooled to room temperature and re‐evacuated to high vacuum (*p* < 5·10^−5^ mbar). Samples subjected to final annealing temperatures of 673 and 873 K are referred to as VA673 and VA873, respectively.

### 2.2 Powder Compaction

Powder compaction was performed via cold uniaxial pressing, resulting in regular disk‐shaped specimens. For this purpose, the powder was transferred into the cavity of a compaction tool (FTIR Pellet Dies, Specac, 13 mm) and uniaxially compressed with a hydraulic press (Atlas Manual Hydraulic Press 15T, Specac). A pressing force of 9.81 kN (equivalent to a load of 1 t) was applied and maintained for 1 min to obtain mechanically stable green compacts. This corresponds to an applied pressure of *p* = 74 MPa, calculated based on the geometry of the compaction tool used.

### 2.3 Materials Characterization

#### 2.3.1 Electron Paramagnetic Resonance (EPR)

Continuous Wave (CW) EPR measurements were conducted with a Bruker EMX plus‐10/12/P/L X‐band spectrometer, equipped with an EMX^Plus^ standard cavity. The resonant magnetic field values were accurately determined using an NMR teslameter. A Suprasil quartz glass tube (*d*_o_ = 5 mm, *d* *_i_* = 3 mm) containing ZnO nanoparticle powders was connected to a high vacuum line with base pressures as low as *p* < 10^−5^ mbar. This setup allows the addition of pure gases (O_2_) and annealing in controlled atmospheres, including high vacuum conditions (*p* < 10^−5^ mbar). EPR spectra were recorded at 100 K under continuous pumping at high vacuum conditions (*p* < 10^−5^ mbar) using a variable temperature unit (Bruker). Typical acquisition parameters included a microwave power of 1 mW, a field modulation frequency of 100 kHz, and a modulation amplitude of 0.2 mT.

To quantify the spins, the Number of Spins Calculation procedure in the Bruker Xenon software was utilized. This method allows the direct determination of the number of unpaired spins in the sample without the need for a reference standard. The underlying equation is shown in Equation (1).

(1) $$N_{S}=\frac{DI\cdot V}{P\cdot B_{m}\cdot Q\cdot c\cdot S\cdot (S+1)\cdot n_{B}\cdot f(B_{1},B_{m})}$$

The acquisition parameters—microwave power P, modulation amplitude $B_{m}$, and resonator Q‐factor Q—are specific to each measurement and automatically stored by the software. Resonator properties, including the calibration factor c and field profile $f(B_{1},B_{m})$, are also accounted for in the software and had been determined during factory calibration using a known standard. The Boltzmann factor $n_{B}$ is computed based on the sample temperature and microwave frequency, both of which depend on the measurement and are recorded by the software. The double integral DI is determined through a two‐step integration of the analyzed EPR signal. Electron spin S and sample volume V must be manually input by the user in the software's processing dialog. The sample volume V is determined by the inner diameter of the EPR tube and the sample length.

#### 2.3.2 UV–Vis–NIR

UV–vis–NIR diffuse reflectance spectra were recorded using a double‐beam Perkin Elmer Lambda 750 UV–vis–NIR spectrophotometer, equipped with a 60 mm Spectralon integrating sphere and two detector units. A R955 PMT detector was used for the ultraviolet and visible range (200–860 nm), while an InGaAs detector covered the near‐infrared region (860–2500 nm). Measurements were performed with a spectral resolution of 2 nm, using a Spectralon diffuse white plate standard as a reference. The acquired reflectance spectra were converted to absorption spectra using the Kubelka–Munk‐transformation. Nanoparticle powders and compacts were measured using dedicated vacuum tight silica cells, comprising a Suprasil optical cuvette and a quartz glass chamber capable of withstanding thermal annealing up to 1173 K. These cells also allow optical measurements under high vacuum conditions or in controlled gas atmospheres. All measurements were conducted at room temperature in a 100 mbar oxygen atmosphere to suppress surface exciton related luminescence in the UV–vis–NIR spectra [32].

### 2.4 Computational Methods

We performed density functional theory (DFT) calculations using the VASP code [33, 34, 35], relaxing structures and for the stoichiometric case also the cell dimensions at the PBE level of theory [36], followed by an evaluation of the energy at the HSE06 level of theory [37], starting from PBE wavefunctions. This approach is needed to correctly describe the electronic structure and especially the bandgap of ZnO, which is severely underestimated using PBE. For PBE calculations, wavefunctions were expanded in planewaves up to a kinetic energy cutoff of 520 eV, which was reduced to 400 eV for HSE06 calculations. In all cases, projector‐augmented wave (PAW) [35, 38] potentials with Zn(3d, 4s), O(2s, 2p), and H(1s) valence states were used. For calculations under pressure, we applied an isostatic stress of 0.74 kbar and reoptimized the lattice parameters (without pressure: *a* = 3.2864 Å, *c* = 5.3070 Å, with pressure: *a* = 3.2857 Å, *c* = 5.3062 Å).

Defect calculations were performed in a 3 × 3 × 2 supercell of the hexagonal unit cell, for which lattice parameters were fixed to describe the dilute defect limit and for which reciprocal space was sampled using a 2 × 2 × 2 gamma‐centered mesh. Defect formation energies were calculated as [39, 40]

(2) $$E_{\mathrm{form}}^{q}=E_{\mathrm{def}}-E_{\text{perf}}-\sum_{i}n_{i}{µ}_{i}+q\left(E_{\text{Fermi}}+E_{\mathrm{VBM}}\right)+\text{Δ}E_{\text{corr}}$$

where, *E*_def_ and *E*_perf_ stand for the DFT energy of the defective and stoichiometric cell, the following term accounts for added (*n*_*i*_ > 0) or removed (*n*_*i*_ < 0) species at a chemical potential *µ*_*i*_. Chemical potentials constrained to the stability range of ZnO with respect to Zn, ZnOH_2_, O_2_, H_2_O, and H_2_, within which we chose Zn‐rich and O‐poor as well as H‐rich conditions to mimic experimental synthesis conditions. The charge state *q* of the defect was controlled by exchanging *q* electrons with a reservoir at an energy of *E*_Fermi_ relative to the valence band (*E*_VBM_), while the last term aligns the electrostatic potential of charged and neutral cells and accounts for image‐charge effects [41]. The self‐consistent Fermi energy of the defective system was computed using py‐sc‐fermi [42], assuming a synthesis temperature of 1000 K, the defect concentration of which was assumed to become frozen during the rapid quench.

## 3 Results and Discussion

The two types of ZnO nanoparticle powders investigated were synthesized in the gas phase, i.e. by chemical vapor synthesis (CVS) [19, 29] and by flame spray pyrolysis (FSP). The systematic comparison of their spectroscopic property changes upon powder compaction was used to explore how intrinsic ZnO particle powder properties such as particle size distribution and surface composition influence the evolution of defect chemistry during mechanical compaction.

Particular focus is placed on the fate of a paramagnetic defect, namely the neutral hydroxyl (OH_O_^0^) species substituting O^2−^ ions, which has a low formation energy under the experimental conditions specific to particle synthesis and processing [17], and corresponds to hydrogenated lattice sites.

**FIGURE 1:**
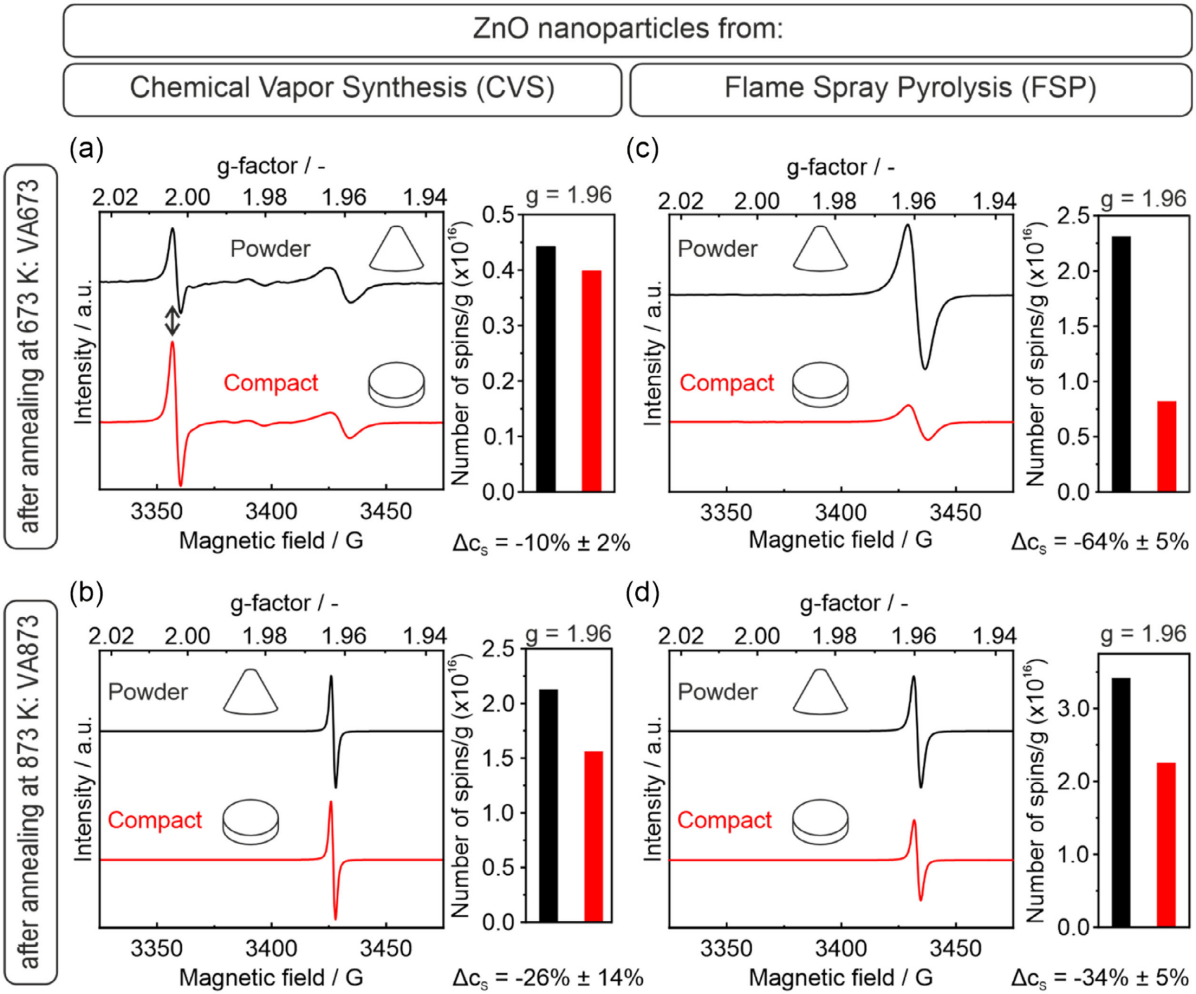
EPR spectra of ZnO nanoparticle derived materials from Chemical Vapor Synthesis (a) CVS‐VA673, (b) CVS‐VA873, and Flame Spray Pyrolysis (c) FSP‐VA673 and (d) FSP‐VA873. Spectra acquired on powders and compacts are plotted in black and red, respectively. The number of spins of the paramagnetic center associated with an EPR signal with a *g*‐value of *g* = 1.96 are provided by the columns. All spectra were acquired at 100 K in dynamic high vacuum (p < 10^−5^ mbar) using a microwave power of 1 mW.

The EPR spectra of the powder samples, serving as reference, alongside those of the compacted materials are shown in Figure 1.

Whereas the spectra in the first row (a) and (c) characterize the paramagnetic properties of VA673 samples treated at temperatures *T* ≤ 673 K in oxygen, the second‐row spectra (b) and (d) correspond to VA873 samples subjected to oxygen treatment at 873 K (VA designates vacuum annealing). The latter annealing step leads to elimination of the isotropic EPR signal at *g* = 2.003 (Double arrow in Figure 1a). Respective resonance is attributed to paramagnetic carbon‐related species (coke), which remain as pyrolysis residues from gas‐phase synthesis on the ZnO nanoparticle surfaces [20, 22, 43]. At temperatures up to 873 K, they convert with O_2_ into CO_2_ and are removed from the ZnO particle surfaces to the gas phase (Figure 1b).

Uniaxial ZnO nanoparticle powder compaction significantly reduces the concentration of OH_O_^0^ defects with their EPR signal at *g* = 1.96^17^. The extent of EPR signal reduction Δ*c*_S_, however, depends on the synthesis route and annealing procedure applied to the ZnO powders prior to compaction: only a modest decrease in spin concentration of Δ*c*_S_ = −10% is determined for the CVS‐VA673 samples (Figure 1a). With Δ*c*_S_ = −26% this decrease is more pronounced for the identical material that is exempt from coke impurities after annealing at 873 K (Figure 1b). Higher intensity reductions are observed for FSP samples (Figure 1c,d), with a decrease of Δ*c*_S_ = −64% and 34% for powders annealed to 673 and 873 K, respectively.

The decrease in OH_O_^0^‐related paramagnetic centers upon compaction is attributed to strain effects and likely originates from tribological interactions that are initiated at particle interfaces.

Density functional theory (DFT) calculations were performed to analyze the microscopic mechanisms and to differentiate between defect charge state transition (i.e., the conversion of paramagnetic into diamagnetic states) and defect annihilation (i.e., the actual elimination of OH_O_ defects). These calculations were performed for thermodynamic parameters, such as the chemical potentials and temperature, consistent with the experimental conditions of gas‐phase synthesis, which include formation temperatures of 1000 K, particle nucleation and growth in oxygen‐deficient and, due to hydrocarbons in the precursor solution and atmosphere, hydrogen‐rich atmospheres. Initially modeled as powders containing unstrained grains, the simulations were then extended to the situation of a compacted powder with an external pressure of 74 MPa and in thermodynamic equilibrium. The Fermi energies were determined using a self‐consistent iterative approach, a standard method in electronic structure calculations to maintain overall charge neutrality.

Local strain subtly modifies both the structural and electronic properties of the system. One notable effect is a shift in the self‐consistent Fermi energy (SC Fermi) from 3.85 eV in the unstrained material to 3.89 eV for the material under strain, i.e., the powder compact. Simultaneously, the formation energies of OH_O_ defects increase under pressure, which would translate into a decrease in their abundance. As summarized in Table 1, the formation energy of the neutral (*q* = 0) OH_O_ increases only slightly from 1.34 eV in the unstrained case to 1.35 eV for the material under strain.

**TABLE 1:** DFT‐derived formation energies for each charge state, corresponding concentrations ($C_{q}$), EPR activity, and relative abundances of hydroxyl groups (OH_O_) substituting oxygen lattice sites. Results are shown for both the unstrained condition (0 MPa, first two rows) and for powders under applied strain (74 MPa, third and fourth row).

Defect	Applied pressure (MPa)	Charge state (*q*)	Formation energy (eV)	Concentration (*C*_q_) (cm^−3^)	EPR active	Relative abundance (%)
OH_O_	0	0	1.34	6.85E + 15	✓	69.8
		+1	1.42	2.97E + 15	✗	30.2
OH_O_	74	0	1.35	6.45E + 15	✓	77.6
		+1	1.46	1.86E + 15	✗	22.4

In contrast, the formation energy of the singly charged state (OH_O_^+^) increases more markedly with lattice strain from 1.42 to 1.46 eV as it is also affected by the change in SC‐Fermi level.

Even though these changes in formation energy may seem tiny, they markedly affect the relative abundance of OH_O_ in the different charge states, the neutral charge state increasing from 70% in the unstrained powder to 78% in the powder compact (Table 1). This conversion to relatively more of the EPR‐active defects is, however, overshadowed by a decrease in their concentration due to the increase in formation energy. The experimentally observed decrease in EPR signal is thus due to the annihilation of OH_O_ defects rather than their conversion to an EPR‐inactive charge state.

With an overall decrease of 16% in the OH_O_ defect concentration, the concentrations of the paramagnetic OH_O_^0^ and diamagnetic OH_O_^+^ defect species decrease by 6% and 37%, respectively (Table 1). Since there are no relevant alternative hydrogen defects (Table S1), we can conclude that powder compaction expels hydrogen from the lattice. This hydrogen removal can be rationalized by the combined effects of strain and local structural disorder introduced during compaction. Local strain destabilizes hydroxylated oxygen lattice sites and renders hydrogen incorporation energetically less favorable, as indicated by our DFT calculations (Table 1). In addition, compaction‐induced disorder may enhance hydrogen mobility by providing diffusion pathways toward the surface. From a chemical perspective, the altered local bonding environment under strain can weaken O—H bonds and thereby promote hydrogen release. Several plausible hydrogen‐removal pathways, including H_2_ formation, water desorption via oxygen vacancy formation, and proton migration followed by recombination, are proposed in the Supporting Information (Equations S1–S4).

The defect annihilation effect predicted by DFT is based on an idealized, thermodynamic equilibrium‐based estimate that is solely derived from changes in formation energies. This approach does not account for non‐equilibrium phenomena that may occur during compaction like local heating, strain‐enhanced defect mobility, or rapid proton diffusion at grain boundaries and surfaces. Such dynamic processes can significantly facilitate the release of hydrogen (see Equations S1–S4 in the Supporting Information), potentially resulting in a larger and more effective OH_O_ defect removal than predicted by the DFT calculations. We further note that our DFT calculations only consider bulk, whereas nanoparticle powders can be significantly affected by surfaces, where defect formation can be altered in the less dense and undercoordinated atomic environment. Such effects will require detailed studies that are a worthy topic of future investigations.

It is unlikely that strain is distributed uniformly throughout the nanoparticle volume. Moreover, the relative surface fraction involved in interparticle contact points within a given powder volume—and thus the strain‐induced defect annihilation—depends on the grain size distribution. For compacts of larger particles—such as powders annealed to 873 K rather than 673 K—the relative surface fraction involved in contacts is reduced. As a result, in such types of particle powders (e.g., FSP VA873), less OH_O_^0^ defects respond to compaction‐induced strain and become annihilated.

**FIGURE 2:**
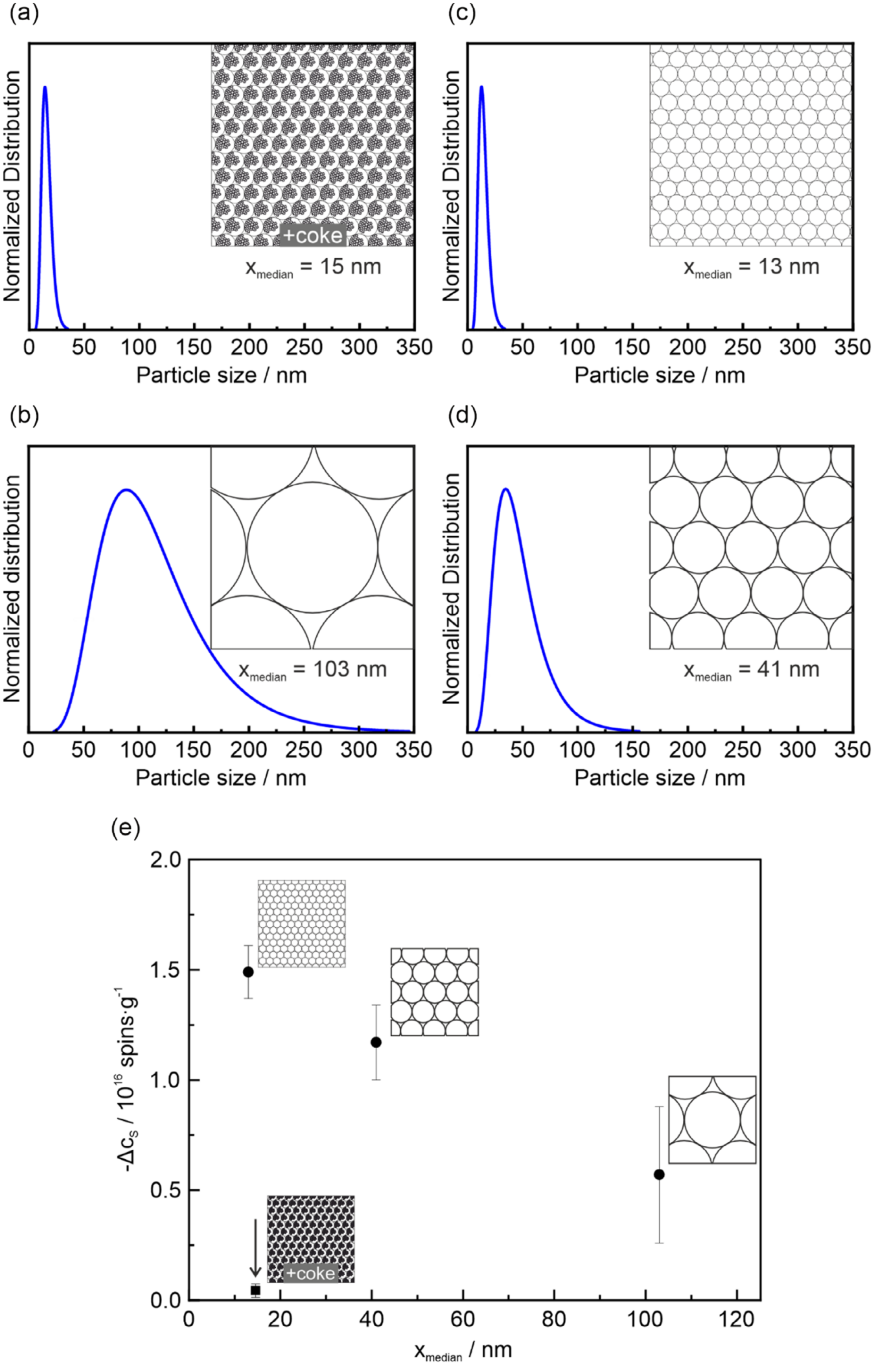
Particle size distributions (blue curves), median particle sizes (d_TEM_) and schematic illustrations of idealized packing of monodisperse spherical particles in a given volume of the ZnO samples: (a) CVS‐VA673, (b) CVS‐VA873, (c) FSP‐VA673, and (d) FSP‐VA873. (e) The experimentally determined decrease of the spin concentration as a result of powder compaction, shown as a function of d_TEM_.

To visualize this tentative trend Figure 2 presents the particle size distributions and the corresponding median particle sizes derived from TEM image analysis [17]. It also provides an idealized schematic illustration of the number of nanoparticles present in a given volume for the ZnO powder samples, assuming monodisperse spherical particles (Figure 2a–d). Figure 2e shows the experimentally observed decrease in spin concentration (–Δ*c*_S_) upon compaction plotted against the median particle size (d_TEM_).

Figure 2e reveals an inverse relationship between particle size and the extent of spin concentration reduction observed for CVS‐VA873 and both FSP ZnO nanoparticle powders upon compaction. Specifically, samples with median particle sizes of 13 nm (FSP‐VA673), 41 nm (FSP‐VA873), and 103 nm (CVS‐VA873) exhibit reductions in spin concentration of Δ*c*_S_ = 1.5·10^16^, −1.2·10^16^, and −0.6·10^16^ spins·g^−1^, respectively (Figure 1b–d). Thus, ensembles of smaller particles are more susceptible to compaction‐induced strain and are consequently more prone to defect annihilation.

The respective data point related to the CVS‐VA673 sample, however, with a significant concentration of surface carbon (Figure 1a), is significantly off this trend (black arrow Figure 2e). Despite its small particle size of 15 nm, the spin concentration decreases by only Δ*c*_S_ = 0.04·10^16^ spins·g^−1^ after compaction (Figures 1a and 2e). The spin concentration of the carbon‐related signal at *g* = 2.003 (Figure 1a) increases by 34% upon compaction, which corresponds to Δ*c*_S_ ≈ +0.05·10^16^ spins·g^−1^ in absolute numbers.

**FIGURE 3:**
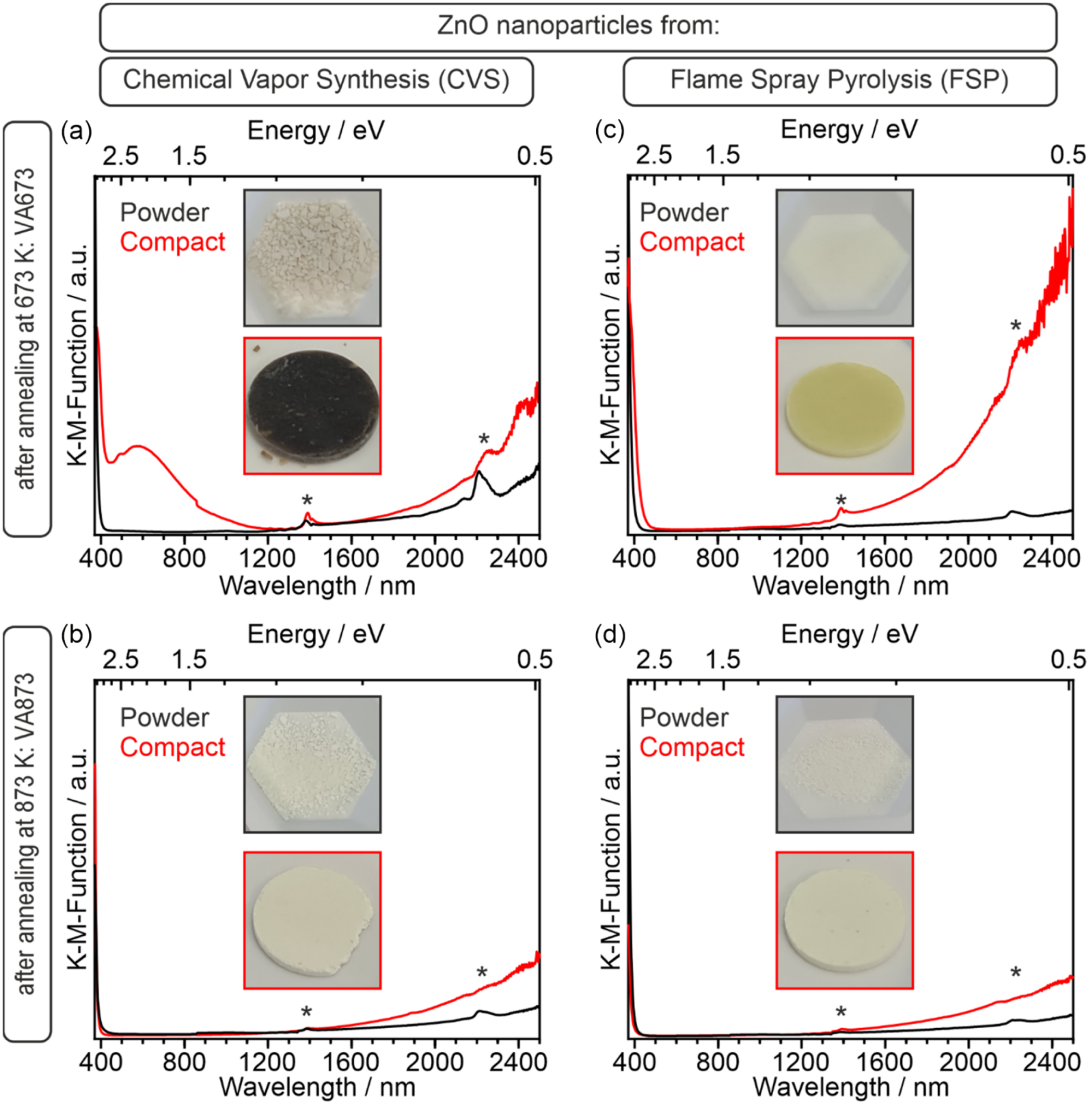
Kubelka–Munk functions determined for ZnO nanoparticle powders (black curves) and corresponding compacts (red curves) obtained from (a) CVS‐VA673, (b) CVS‐VA873, (c) FSP‐VA673, and (d) FSP‐VA873, and recorded by diffuse reflectance at room temperature and in O_2_ atmosphere (*p* = 100 mbar). Insets show digital photographs of the samples investigated. Signal components marked with asterisks denote instrumental artifacts.

Another manifestation of the surface carbon impurities relates to the color change between the powders and powder‐derived compacts, as it was determined by Vis–NIR Diffuse Reflectance (Figure 3a).

After annealing at 673 K (VA673, top row), ZnO nanoparticles produced by CVS and FSP adopt similar particle size distributions and exhibit comparable crystallinity. The compaction‐induced changes of the integral optical ensemble properties between the two samples, however, differ significantly: the CVS‐derived compact turns black and displays a broad absorption in the range of visible light (Figure 3a), whereas the FSP‐derived nanoparticle compact—which is exempt from EPR‐active carbon‐related species—adopts a yellow color (Figure 3c). Both compacts derived from powders annealed to 873 K remain, after surface carbon removal, as white as the starting powders (Figure 3b,d).

The black coloration of the CVS‐VA673 compact is attributed to residual polyaromatic carbon surface impurities (≈5 at.%, as revealed by an earlier Auger electron spectroscopy (AES) analysis [29]). They originate from the chemical vapor synthesis (CVS) process and survive annealing at temperatures below 873 K.

A second important observation that arises from nanocrystal powder compaction is the yellow coloration observed in the FSP‐VA673 compact (Figure 3c), which is an optical manifestation of an absorption edge shift by ≈0.2 eV since this sample absorbs in the blue region of the visible spectrum. Similar effects have been observed on aggregated TiO_2_ nanocrystals [44] and In_2_O_3_ nanocrystals [45]. In the case of In_2_O_3_, the formation of a nanoparticle network from individual nanoparticles leads to a visible color change from slightly yellow to orange [45].

**FIGURE 4:**
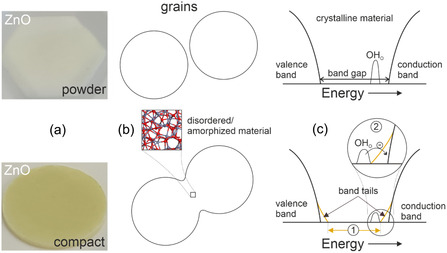
(a) Optical changes that the ZnO nanoparticle powder (top) undergoes upon compaction (bottom). (b) Schematic representations illustrating the emergence of contact regions between grains during compaction, as well as (c) associated changes in the electronic structure. The decrease in the concentration of shallow donor states upon compaction is exaggerated in (c) for better visibility.

Such effects (Figures 3c and 4a) are linked to the emergence of solid–solid interfaces [44, 45]. The structural complexity of the contact region arises not only from strain, but also from the difference in long‐range atomistic order on both sides of the contact interfaces between the grains (Figure 4b). Strain, local deviations from crystallinity, and partial amorphization explain the formation of band tails extending above and below the valence‐ and conduction‐band edges, respectively (Figure 4c).

As a result of this band‐tail formation, electron excitation from the valence band into the conduction band can occur at slightly lower energies than in the nanoparticle powder with a significantly lower concentration of solid–solid interfaces (Effect ➀ in Figure 4c). This accounts for the observed redshift of the absorption edge and the yellow coloration of the FSP‐VA673 compact (Figure 3c).

All four powder compacts display a broad absorption feature in the near‐infrared (NIR) region in their diffuse reflectance spectra (Figure 3). This Drude‐type absorption can be attributed to intraband transitions associated with free charge carriers, originating from the thermal excitation of electrons from shallow donor states, such as OH_O_^+^ centers, into the conduction band [46, 47, 48, 49]. Importantly, the NIR absorption increases upon compaction and is significantly more pronounced in the FSP‐VA673 sample (Figure 3c).

DFT calculations predict a strain‐induced depletion of ionized OH_O_^+^ centers (the primary source of free electrons, Table S1) and neutral OH_O_^0^ centers (involved in defect‐related transitions) upon compaction (Table 1), thereby making changes in the concentration of OH_O_ defects inconsistent with the experimentally observed increase in NIR free‐carrier absorption. DFT further predicts that neutral, diamagnetic oxygen vacancies (V_O_^0^), in which both electrons are localized at the vacancy site and which occur at concentrations comparable to those of OH_O_^0^ defects in ZnO nanoparticle powders [17], introduce deep‐level donor states located approximately 1 eV above the valence‐band maximum. Due to their deep energetic position, V_O_^0^ defects do not act as a source of free charge carriers and therefore cannot contribute to the observed broad NIR absorption.

Hence, changes in the concentration of these defects cannot explain the enhanced NIR absorption. Band tailing (Figure 4c), however, does. The conduction‐band minimum becomes smeared toward lower energies. As a result, electrons can more effectively move from shallow OH_O_ donor states into the conduction band by thermal excitation at room temperature. Thereby, they contribute to Drude‐type absorption (Figure 3 and effect ➁ in Figure 4c). This effect overcompensates for the overall decrease in OH_O_ concentration in the compacts and explains the enhanced NIR absorption upon compaction. Compared to larger particles (Figure 2b,d) the effect is more pronounced for smaller particles (Figure 2c) due to their increased contact‐area‐to‐volume ratio.

With respect to strain‐induced changes in defect chemistry, the carbonaceous surface layer in CVS‐VA673‐derived samples influences both the optical response and the stability of point defects. This represents a key observation in tribochemistry. By absorbing localized stress during powder compaction, this layer limits or even prevents the propagation of strain into the bulk of the grains, effectively passivating the structure. Acting in this way, it explains the negligible defect annihilation in CVS‐VA673 upon compaction (Δ*c*_S_ = −0.04·10^16^ spins·g^−1^) as compared to the FSP‐VA673 sample (Δ*c*_S_ = −1.5·10^16^ spins·g^−1^), despite their similar particle sizes of 15 and 13 nm (Figures 1 and 2). The combined effects of residual carbon, structural disorder, and strain‐driven defect evolution provide important insights into the tunable electronic and optical properties of ZnO nanoparticle‐based materials.

Comparing these results with our previous study on cold‐sintered ZnO [18] shows that in both cases the defect chemistry of ZnO is modified, as reflected by intensity changes of the paramagnetic signal at *g* = 1.96, which is associated with hydroxylated oxygen lattice sites. However, the direction (intensity increase or decrease) of this change differs significantly between the two processing routes. Mechanical compaction leads to a reduction of the *g* = 1.96 signal, indicating that hydrogen incorporation becomes less favorable and that hydrogen is at least partially removed from the lattice under compressive strain. In contrast, cold sintering in the presence of hydrogen‐containing transient phases promotes hydrogen incorporation, resulting in an increased *g* = 1.96 signal. This intensity increase is more pronounced in acidic solutions than in pure water [18]. While a detailed atomistic understanding of these contrasting responses would require dedicated DFT calculations and targeted experiments, the present findings clearly demonstrate that mechanical compaction and cold sintering induce qualitatively different hydrogen‐related defect behavior. This highlights the role of the chemical environment and processing conditions in governing ZnO defect chemistry, in addition to mechanical effects.

## 4 Conclusion

This combined experimental and theoretical investigation demonstrates that mechanical compaction of ZnO nanoparticle powders significantly modifies the defect chemistry and optical properties of the grains inside the derived compacts. EPR spectroscopy revealed a strain‐induced decrease in the concentration of paramagnetic OH_O_^0^ defects resonating at *g* = 1.96, indicating reduced stability of hydrogenated lattice sites under compressive stress.

Complementary Vis–NIR diffuse reflectance spectroscopy measurements revealed enhanced near‐infrared (NIR) absorption for all samples and changes in absorption in the visible spectrum for samples annealed to 673 K upon compaction. The observed optical changes, together with the reduction in paramagnetic centers, are influenced by non‐uniform strain distribution inside the powders, which is subject to number and area of particle contacts and varies with particle size and surface composition of the grains.

Consistent with experiment, DFT calculations reveal that stress destabilizes protonated oxygen sites, leading to hydrogen release and changes in defect concentrations.

Mechanical compaction of ZnO nanostructures in contact and the associated emergence of compressive strain at contact points and asperities—often accompanied by partial amorphization at particle interfaces—can significantly affect the optoelectronic properties of ZnO nanoparticles. This study emphasizes the importance of strain effects in the design and processing of particle‐based functional materials and, in particular, those with piezoelectric and photocatalytic activity.

## Supporting Information

Additional supporting information can be found online in the Supporting Information section. **Supporting Fig. S1:** Schematic illustration of the thermal annealing protocol for ZnO nanoparticles with a final annealing temperature of a) 673 K (VA673) and b) 873 K (VA873). **Supporting Table S1:** Concentrations of free electrons in the conduction band, OH_O_^+^ defects, and H_O_^+^.

## Author Contributions

**Korbinian Aicher**: investigation, formal analysis, data curation, writing – original draft. **Thomas Berger**: formal analysis, data curation, writing, review & editing. **Antonios Litovoilis**: investigation, formal analysis, data curation. **Ulrich Aschauer**: formal analysis, review and editing. **Oliver Diwald**: supervision, funding acquisition, conceptualization, writing – original draft, review & editing.

## Funding

This study was supported by Austrian Science Fund (FWF) [10.55776/P 34906].

## Conflicts of Interest

The authors declare no conflicts of interest.

## Supporting information

Supplementary Material

## Data Availability

The data that support the findings of this study are available from the corresponding author upon reasonable request.
