# Supplementary material for: Strain‐Induced Redistribution of Point Defects in ZnO Nanoparticles
Source: Small Sci. 2026 Mar 23;6(3):e202500642. doi: 10.1002/smsc.202500642 (PMC13097541; doi:10.1002/smsc.202500642)
Supplement: Supplementary file 1 — Supplementary Material [file SMSC-6-e202500642-s001.pdf]

# Strain-induced Redistribution of Point Defects in ZnO Nanoparticles

*Korbinian Aicher*[a], *Thomas Berger*[a], *Antonios Litovoilis*[a,b], *Ulrich Aschauer*[a,*] *and Oliver Diwald*[a,*]

[a] Department of Chemistry and Physics of Materials,

Paris-Lodron University Salzburg, Jakob-Haringer-Straße 2a,

A-5020 Salzburg, Austria

[b] Present address: AMOLF, Science Park 102, 1098 XG, Amsterdam,

The Netherlands

E-mail: oliver.diwald@plus.ac.at

ulrichjohannes.aschauer@plus.ac.at

**Keywords**: particles in contact, intralattice hydrogen, flexoelectronics, coke

# 1 Supporting Information – Materials and Methods

*Thermal annealing:*

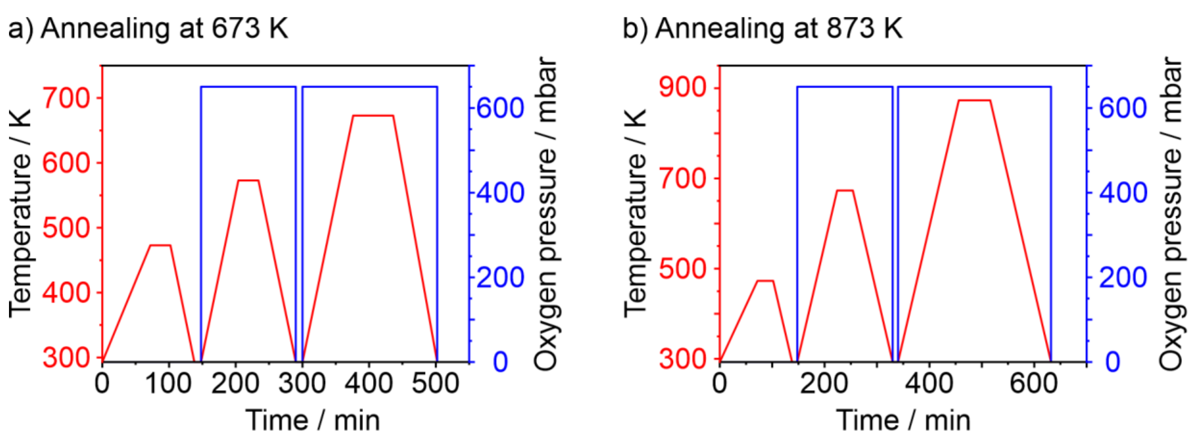


Figure S1: Schematic illustration of the thermal annealing protocol for ZnO nanoparticles with a final annealing temperature of a) 673 K (VA673) and b) 873 K (VA873).

# 2 Supporting Information – Results and Discussion

Table S1: Concentrations of free electrons in the conduction band, $OH_O^+$ defects, and $H_O^+$.

| Species | Concentration @ 0 MPa [$10^{15}\cdot cm^{-3}$] | Concentration @ 74 MPa [$10^{15}\cdot cm^{-3}$] |
|---|---|---|
| Free electrons | 3.06 | 1.92 |
| $OH_O^+$ | 2.97 | 1.86 |
| $H_O^+$ | 0.09 | 0.06 |

**Mechanisms of hydrogen removal from the ZnO lattice:**

We propose that hydrogen is removed from the system via one or more of the following mechanisms: molecular hydrogen formation (Equation S1), water desorption via oxygen vacancy formation (Equation S2), or proton migration (Equation S3) followed by recombination into $H_2$ (Equation S4):

$$2O - H^{\cdot} \rightarrow 2\,O^{2-} + \mathrm{H}_2(g) \quad \text{Equation S1}$$

$$2O - H^{\cdot} \rightarrow O^{2-} + \mathrm{V_O} + \mathrm{H_2}O(g)$$ Equation S2

$$O - H^{\cdot} \rightarrow O^{2-} + \mathrm{H}^{+} + e^{-}$$ Equation S3

$$2H^{+} + 2e^{-} \rightarrow \mathrm{H_2}(g)$$ Equation S4
